# Liver–joint axis: Hepatitis B virus as a contributor to rheumatoid arthritis pathogenesis

**DOI:** 10.1080/21505594.2025.2590267

**Published:** 2025-11-14

**Authors:** Hui Wu, Na Zhao, Xiaoyu Zhang, Yi Zhang, Hongxing Wang

**Affiliations:** a Department of Clinical Laboratory, Qilu Hospital of Shandong University, Jinan, Shandong, China; b Department of Clinical Laboratory, Qilu Hospital of Shandong University (Qingdao), Qingdao, Shandong, China; c Shandong Engineering Research Center of Biomarker and Artificial Intelligence Application, Jinan, Shandong, China; d Department of Laboratory Medicine, The 960, Hospital of the PLA Joint Logistics Support Force, Jinan, Shandong, China; e Shandong Key Laboratory of Medicine and Prevention Integration in Rheumatism and Immunity Disease (PKL2024C19), Jinan, Shandong, China

**Keywords:** Hepatitis B virus, rheumatoid arthritis, liver-joint axis, fibroblasts, metabolites

## Abstract

Hepatitis B virus (HBV) is increasingly recognized for its involvement in extrahepatic diseases, including rheumatological manifestations such as arthritis and joint pain. This review introduces the concept of the liver-joint axis, hypothesizing that HBV may contribute to rheumatoid arthritis (RA) pathogenesis through immune and metabolic dysregulation. We emphasize the effect of HBV infection on fibroblast activation, metabolic reprogramming, and Th17/Treg imbalance. Transcriptome analysis further elucidates the complex signaling networks underlying HBV-associated RA. These findings support a pathogenic role for HBV in joint inflammation and suggest novel therapeutic opportunities for targeting HBV-driven RA.

## Introduction

Hepatitis B virus (HBV), a member of the Hepadnaviridae family [1], possesses a 3.2 kb partially double-stranded DNA genome that encodes four overlapped open reading frames (ORFs): preC/C, preS/S, P, and X [2]. HBV is transmitted through vertical (mother-to-child), blood transfusion, and sexual routes. Infection with HBV contributes to a spectrum of liver diseases, ranging from acute and chronic hepatitis to cirrhosis and hepatocellular carcinoma (HCC) [3]. Additionally, HBV has also been implicated in extrahepatic manifestations, including polyarthralgia and polyarthritis in rheumatological conditions [4]. However, the pathogenic mechanisms remain poorly understood. Our recent study demonstrated that HBV-encoded X protein (HBx) facilitates the transition of fibroblasts to myofibroblasts within the rheumatoid microenvironment, suggesting a potential mechanistic link between HBV infection and rheumatoid arthritis (RA) pathogenesis [5].

RA is a chronic systemic autoimmune disease characterized by persistent synovitis, widespread inflammation, and disease-specific autoantibodies. Although its etiology remains incompletely understood, RA is believed to arise from a complex interplay of genetic, epigenetic, and environmental factors [6]. Viral infections, especially HBV, have drawn more attention as environmental factors that may initiate or modify autoimmune reactions. Evidence suggests that HBV infection may influence immune regulation beyond the liver, potentially establishing a pathological crosstalk between the hepatic and articular tissues.

This review synthesizes recent advances in understanding the interplay between HBV infection and RA. We propose the novel concept of a liver-joint axis, whereby HBV may exacerbate both hepatic and rheumatic manifestations through immune and metabolic pathways. This framework provides novel insights into the mechanisms of viral infection and chronic inflammation, which may inform future therapeutic strategies targeting HBV-associated extrahepatic diseases.

## HBV-RA crosstalk

The relationship between the liver and joints is intricate and bidirectional. Patients with primary hepatic disorders often exhibit extrahepatic manifestations, such as joint involvement, seen in viral arthritis associated with hepatitis viruses, autoimmune liver diseases, and metabolic dysfunctions [7]. Notably, acute HBV infection may initially manifest with arthritis-like symptoms, such as swelling and pain in the metacarpophalangeal, metatarsophalangeal, and interphalangeal joints, closely mimicking RA [8]. Although overt synovitis and joint damage are uncommon, approximately 25% of individuals with chronic HBV infection experience arthritic symptoms [9]. Patients with RA show a higher incidence of autoimmune liver diseases, including primary biliary cirrhosis (PBC), autoimmune hepatitis (AIH), and primary sclerosing cholangitis (PSC) [10,11]. Furthermore, immunosuppressive therapies commonly used in RA, such as biologic/targeted synthetic disease-modifying antirheumatic drugs (b/tsDMARDs), heighten the risk of HBV reactivation (HBVr), particularly in those with chronic HBV infection [12,13].

HBV infection remains a significant global health burden. Despite the availability of an effective vaccine, over 290 million people have chronic HBV infections, and approximately 820,000 people die from HBV-related complications [14,15]. Meanwhile, RA affects approximately 0.5–1% of the global population, with a markedly higher incidence in women, occurring two to three times more frequently than in men, and a mean age of onset of around 50 years [16]. The association between HBV and RA remains controversial. Although some studies have reported no association [17], others have shown an increased prevalence and risk of HBV infection in RA patients [18,19]. For example, the HBsAg positivity rate among RA patients in China was 11.2%, compared to 8.7% in age-matched controls [20].

Additionally, hepatitis B core antigen (HBcAg) has been detected in the synovium of up to 64% of RA patients with chronic HBV, suggesting a possible pathogenic role for HBV in RA [21]. Conversely, RA patients are at increased risk of HBVr during treatment with antirheumatic or immunosuppressive drugs [12,13,22,23], which can lead to severe outcomes such as fulminant hepatitis, liver failure, or cirrhosis. Therefore, pre-treatment screening for HBV serological markers, including HBsAg, anti-HBc, and anti-HBs, is essential before initiating immunosuppressive therapy in RA patients [24]. Furthermore, comprehensive, multi-regional epidemiological studies are necessary to further clarify the relationship between HBV infection and RA. Such investigations will contribute to the development of integrated, multidisciplinary management approaches and more effective monitoring strategies for patients with concurrent RA and HBV infection.

## Mechanisms underlying the liver-joint axis

Contemporary research increasingly recognizes that several disease processes are mediated through inter-organ axes, such as the gut-lung axis [25] and the lung-brain axis [26,27]. Among these, the gut-joint axis has garnered particular attention in RA pathogenesis. Similarly, in RA-associated interstitial lung disease (RA-ILD) [28], two potential mechanistic pathways have been proposed: joint-to-lung or lung-to-joint propagation [27]. These findings highlight how immune signaling, microbial metabolites, and systemic inflammation can create bidirectional axes between seemingly unrelated organs.

The liver-joint axis constitutes a novel pathological framework, with HBV as a potential mechanistic link. In addition to its hepatotropism, HBV is implicated in extrahepatic autoimmune disorders. We propose that HBV may act as a critical mediator of liver-joint crosstalk, supported by four lines of evidence.

## Fibroblast

**Figure 1.**
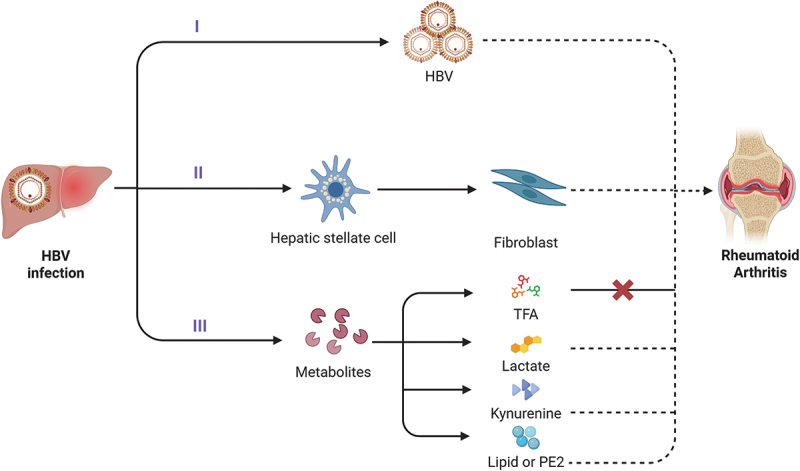
Hypothesized mechanisms through which HBV may contribute to RA progression. (I) HBV may directly access the synovium and influence the progression of RA. (II) HBV-induced activation of hepatic stellate cells may differentiate into fibroblast phenotype, facilitating the migration and proliferation of synovial fibroblasts. (III) HBV infection may alter systemic metabolite profiles, thereby contributing to RA pathogenesis. The red cross indicates the suppressive effect of TFA on RA-related processes. Dashed lines represent hypothetical pathways pending experimental validation. Created with Biorender.com.

Fibroblast is essential for maintaining tissue architecture and orchestrating repair processes. However, in both HBV infection and RA, it emerged as a pivotal effector of disease progression. Hyperactive fibroblast-like synoviocytes (FLSs) overproduce extracellular matrix (ECM) proteins, resulting in joint inflammation and damage [29]. HBV infection dysregulates fibroblast activity both systemically and within hepatic tissue. A well-characterized example is the activation of hepatic stellate cells (HSCs), which differentiate into myofibroblasts and contribute to liver fibrosis [30]. However, the role of HSCs in HBV-associated RA remains unclear. Specifically, whether HSCs can differentiate into circulating fibroblasts that migrate to the synovium and contribute to joint pathology requires further investigation. Intriguingly, HBV has been detected in the synovial fluid of RA patients [31], suggesting a possible direct interaction between HBV and FLSs (Figure 1).

## Metabolites

HBV infection regulates the host metabolic network to generate key metabolites crucial to the pathogenesis of HBV-RA. Through reprogramming host glucose, amino acid, and lipid metabolism pathways, HBV promotes the accumulation of pro-inflammatory metabolites (e.g. lactate, kynurenine, PGE_2_) while suppressing protective metabolites like TFA regulatory functions. Metabolic dysregulation ultimately contributes to the development of RA by disrupting the “metabolism-immunity” network. Targeting metabolic nodes (such as HK2, IDO1, and LXR) or exogenously supplementing TFA may offer novel therapeutic avenues for the prevention and treatment of HBV-related RA (Figure 1).

## Trans-ferulic acid

Although HBV is a risk factor for RA, only a subset of exposed individuals ultimately develops clinical symptoms. Trans-ferulic acid (TFA) is a common phenolic compound with broad biological activities. It has been reported to exert anti-fibrotic effects by suppressing the TGF-β/Smad signaling pathway and consequently reducing extracellular matrix production [32]. Moreover, metabolomic analysis in HBV-CIA mice revealed reduced levels of TFA in the liver, plasma, and synovial fluid compared with uninfected controls [5]. Functional assays demonstrated that TFA inhibited the effects of TRAFD1 overexpression in human FLS (HFLS) by downregulating FMT-associated proteins. In vivo, TFA treatment alleviated arthritis symptoms in HBV-CIA mice via the inhibition of TRAFD1. Collectively, TFA may be a critical negative regulator of the HBV-TRAFD1 axis, positioning it as a potential preventive agent against RA development in HBV-infected individuals [5].

## Lactate

Lactate, a major metabolite produced at the end of glycolysis under anoxic conditions, regulates immune cell differentiation and function [33]. For instance, lactate promotes IL-17 production and the differentiation of Th17 cells through the STAT3/RORγt axis, which exacerbates cartilage destruction and bone erosion [34]. It may also drive macrophage polarization toward the pro-inflammatory M1 phenotype, thereby disturbing the balance between M1 and M2 macrophages [35]. Synovial hypoxia drives lactate accumulation, which increases arthritis inflammation and diminishes therapeutic effectiveness [36–38]. Recent evidence further indicates that quiescent FLS from RA patients (RA-FLSs) produce significantly higher levels of lactate than their osteoarthritic counterparts (OA-FLSs) [39]. HBV infection is known to reprogram host cellular metabolism, including glucose metabolism. Specifically, HBV induces aerobic glycolysis via upregulation of hexokinase 2 (HK2), leading to excess lactate production [40,41]. Collectively, these data implicate HBV-induced lactate accumulation as a potential driver of synovial inflammation and metabolic dysregulation in RA.

## Tryptophan

Evidence for the role of tryptophan metabolism, including the kynurenine (Kyn), 5-hydroxytryptamine (serotonin, 5-HT), and indole pathways, in RA has existed for decades. Moulin et al. have found that disease activity and pro-inflammatory markers in RA patients are positively correlated with kynurenine [42]. Similarly, metabolites of the kynurenine (Kyn) pathway contribute to the progression of systemic lupus erythematosus (SLE) [43]. Indoleamine 2,3-dioxygenase 1 (IDO1), the rate-limiting enzyme in tryptophan catabolism, catalyzes the conversion of tryptophan to kynurenine. Elevated IDO1 activates mTOR signaling in T cells, skewing differentiation toward Th17 cells while restraining Treg cells [44]. During HBV infection, immune cell activation triggers the production of interferon-γ (IFN-γ), which subsequently upregulates IDO1expression in epithelial cells and dendritic cells [45]. Hence, we hypothesize that HBV may aggravate synovial inflammation in RA by modulating the kynurenine pathway.

## Lipid metabolism

Lipid metabolism has recently emerged as a critical regulator of inflammation and immunity. For example, cholesterol accumulation induces endoplasmic reticulum (ER) stress, which activates the inflammasome to promote IL-1β production [46]. Among the key mediators of lipid metabolism, liver X receptors (LXRs), comprising LXRα and LXRβ, are ligand-activated transcription factors that regulate intracellular cholesterol and lipid homeostasis [47]. LXR activation, driven by elevated cholesterol levels, has also been shown to exacerbate eosinophilic airway inflammation by enhancing IL-5 and IL-13 production in T cells [48]. HBV-encoded X protein (HBx) enhances the expression and transcriptional activation of LXRα/β, thereby promoting hepatic lipid synthesis [49–51]. Interestingly, LXR is markedly upregulated in synovial macrophages from RA patients [52]. Moreover, activation of LXRα/β significantly exacerbates disease onset and severity in a murine model of collagen-induced arthritis (CIA) [53]. Therefore, HBV infection may exacerbate RA pathogenesis by HBx-dependent activation of LXRα/β, which orchestrates synovial macrophage lipid metabolism reprogramming and inflammatory responses.

## Prostaglandin E2

Prostaglandin E2 (PGE2), the most abundant bioactive lipid mediator in humans, regulates a wide array of physiological processes, including inflammation, blood pressure, fertility, and bone homeostasis [54]. It acts by binding to specific E-type prostaglandin (EP1-EP4) receptors on plasma or organelle membranes. The COX-2/PGE2/EP4 signaling pathway promotes bone formation and strength, whereas the COX-2/PGE2/EP3 pathway facilitates cardiac repair by promoting the secretion of vascular endothelial growth factor (VEGF) [55]. In RA, PGE2 has been associated with edema and the erosion of cartilage and juxta-articular bone, suggesting its role in disease pathogenesis [56]. In an animal model of polyarthritis, neutralizing PGE2 with monoclonal antibodies attenuated disease progression [57]. In addition, therapeutic strategies targeting PGE2, whether through inhibition of its biosynthetic enzymes, modulation of its in vivo levels, or disruption of downstream signaling pathways, represent a rational and promising approach to RA management [58]. Higher serum PGE2 levels have been observed in chronic HBV patients compared to healthy subjects, with concentrations directly proportional to both liver damage and viral load [59]. Therefore, HBV infection may promote RA progression by affecting PGE2 levels.

## Signal pathways

The pathogenicity of HBV is closely linked to the activation of multiple host cell signaling pathways. It is known that HBV-encoded proteins contribute to disease progression by engaging key molecular cascades, including the Wnt/β-catenin, PI3K/AKT, MAPK, JAK/STAT, and NF-κB pathways [60–65]. Intriguingly, these identical pathways are also implicated in RA disease progression. For example, both JAK/STAT and NF-κB signaling pathways contribute to bone destruction in RA patients [66,67].

**Figure 2.**
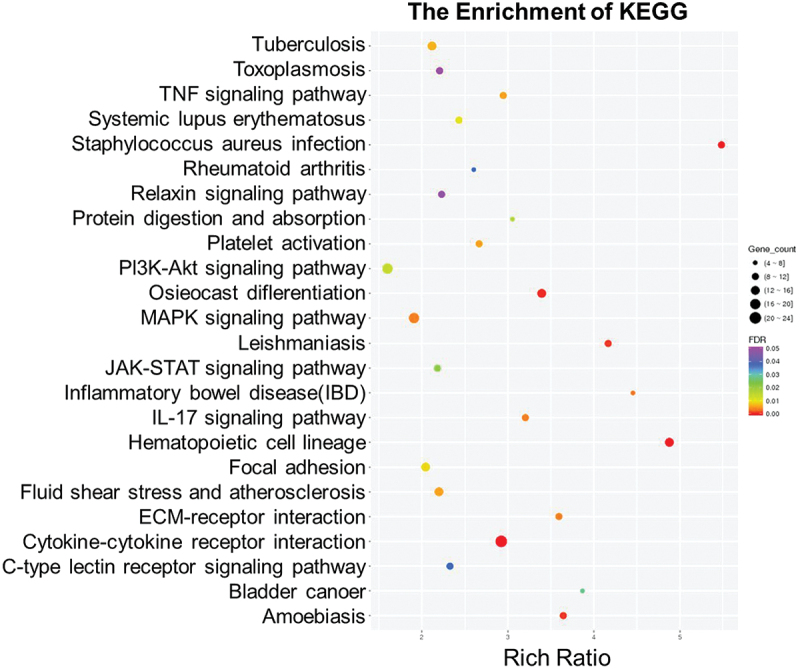
Transcriptome analysis of CHB-RA and CHB patients screened out the common signaling pathway.

To further investigate the molecular mechanisms underlying HBV-associated RA, we conducted transcriptomic profiling of peripheral blood mononuclear cells (PBMCs) from individuals with chronic hepatitis B (CHB) who had concomitant RA or not (https://doi.org/10.6084/m9.figshare.29558216). Pathway enrichment analysis identified significant dysregulation in immune-inflammatory networks, which we categorized into three major functional clusters (Figure 2): autoimmune and inflammatory pathways (Rheumatoid arthritis, TNF signaling, IL-17 signaling, Systemic lupus erythematosus, and Inflammatory bowel disease), infectious disease-related responses (Tuberculosis, Staphylococcus aureus infection, Leishmaniasis, Toxoplasmosis), and cell proliferation and tissue remodeling pathways (PI3K/AKT signaling, MAPK signaling, JAK/STAT signaling, Focal adhesion, ECM-receptor interaction, and Osteoclast differentiation). These findings underscore the complexity of signaling networks involved in HBV-associated RA and provide potential molecular targets for therapeutic intervention.

## Immune modulation

Infectious diseases are shaped by a complex interplay between the host and the invading pathogen. In HBV infection, adaptive immune cells, particularly T and B lymphocytes, are central to both the failure to eliminate the virus and liver inflammation. Extensive studies have demonstrated that CD4^+^ T cells play a pivotal role in orchestrating the host defense response to HBV. Furthermore, the interaction between FLSs and innate immune cells (e.g. macrophages, dendritic cells, mast cells, and natural killer cells) and adaptive immune cells (e.g. B and T lymphocytes) constitutes a critical driver of RA progression.

## CD4+ T cells

The Th17/Treg cell imbalance is a recognized risk factor for HCC progression in patients with HBV infection and also plays a critical role in driving joint inflammation [68,69]. Clinical studies have shown that Th17 cells are significantly elevated in patients with chronic HBV infection or HBV-related liver failure compared to healthy controls [70]. In the liver microenvironment, Th17-derived IL-17 cooperates with IL-6 and TNF-α to activate hepatic stellate cells and drive fibrogenesis. Similarly, RA is characterized as a Th17-driven immune disorder [71], where IL-17 in the synovium contributes to synovitis, pannus formation, and progressive cartilage and bone destruction [72,73]. Interestingly, Th17 cells may also mediate liver pathology secondary to joint inflammation. Yi et al. reported that hemarthrosis induces Th17 differentiation, which contributes to liver disease [74].

The immunoregulatory function of Treg cells has been well characterized throughout HBV infection. In chronic hepatitis B (CHB), Treg cells are markedly increased, where they suppress virus-specific T cell responses, secrete anti-inflammatory cytokines such as IL-10 and TGF-β, and promote immune tolerance to HBV, thereby facilitating viral persistence [75]. Given their immunosuppressive properties, Treg cells may paradoxically attenuate autoimmune responses in RA, particularly in patients with chronic HBV infection.

## Macrophage

Our understanding of how macrophages transition between phenotypes to inhibit viral replication and modulate virus clearance remains limited. As liver tissue-resident macrophages, Kupffer cells (KCs) potentially play a pivotal role in the regulation of HBV infection. They may achieve this by directly suppressing viral replication via cytokine production or indirectly through interactions with other immune or parenchymal cells [70]. It is noteworthy that, in HBV infection, KC-derived cytokines (including IL-8, IL-10, and IL-12) exhibit pleiotropic effects, some of which paradoxically may exacerbate RA progression despite their antiviral properties.

## Nk cells

Natural killer (NK) cells are key components of the innate immune system, playing an essential role in antiviral defense and immune regulation. Although some studies suggest a passive role for NK cells, accumulating evidence supports their active involvement in disease pathogenesis. Notably, a positive correlation between activated NK cells and serum alanine aminotransferase (ALT) levels implicates NK cell activation in hepatic necroinflammation during acute HBV infection [76]. We previously demonstrated that CD38^+^ NK cells contribute to the progression of RA by modulating CD4^+^ T cell subsets [77]. Moreover, HBV-induced activation of neutrophils can trigger the release of reactive oxygen species, leading to aberrant expression of CD160 in NK cells, an event that may play a critical role in RA pathogenesis.

**Figure 3.**
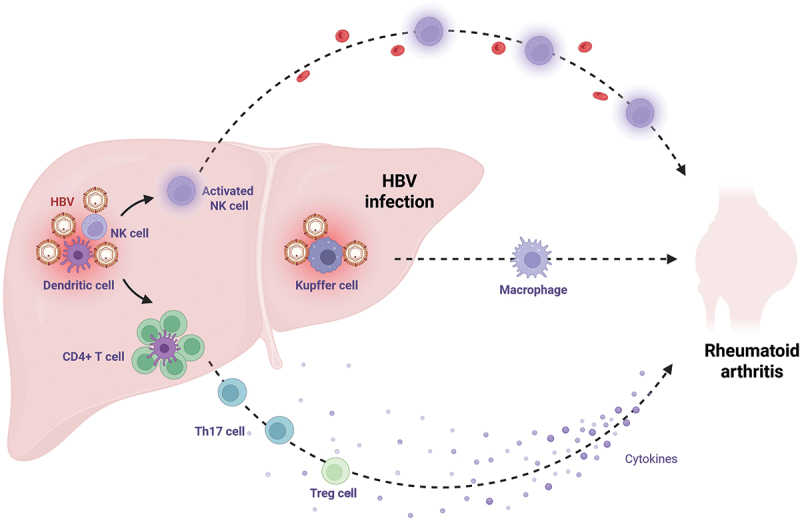
HBV-mediated modulation of immune cell populations in rheumatoid arthritis. HBV may influence the activation, differentiation, or function of key immune cell subsets involved in RA pathogenesis, including T cells, macrophages, and DC cells. Dashed lines represent hypothetical pathways pending experimental validation. Created with Biorender.com.

In summary, HBV may contribute to the progression of RA by disrupting the balance between Th17 and Treg cells. This immune imbalance extends to the activation and function of macrophages, neutrophils, and NK cells, collectively reshaping the cytokine environment. Key cytokines affected include interleukin-10 (IL-10), transforming growth factor-β (TGF-β), interleukin-17 (IL-17), IL-6, IL-1, and IL-8, all of which play central roles in RA pathogenesis (Figure 3).

## Implications for prevention and therapy

Our prior research identified Helicobacter pylori (*H. pylori*) infection as a contributing factor in RA progression, suggesting that eradication therapy could represent a viable strategy for disease management [78]. Analogously, if HBV is verified as a driver of RA pathogenesis, antiviral therapy may constitute a core component of treatment for HBV-associated RA.

## Current therapy

Current first-line therapies for chronic HBV infection include nucleos(t)ide analogues (NAs) and pegylated interferon alfa (PEG-IFNα). However, the effects of HBV therapies on patients with RA remain largely unexplored. To date, only one case report describes an RA patient who developed HBV reactivation and was treated with the NA entecavir (ETV), leading to a reduction in serum erythrocyte sedimentation rate (ESR), a clinical indicator of disease activity [79]. This observation suggests the potential therapeutic relevance of HBV-targeted treatments in RA.

## Novel therapeutic targets

Recent research into HBV-associated joint and liver pathology has identified several novel molecular candidates for therapeutic intervention. One study highlighted the critical role of the RUNX2/ITGBL1 axis in the activation of hepatic stellate cells and fibrotic remodeling, suggesting it is a promising target for anti-fibrotic therapy [80]. Modulating this pathway may simultaneously affect synovial fibroblast activity, offering potential therapeutic benefits for both hepatic and articular fibrosis. Additionally, our research demonstrated that TFA treatment mitigates HBV-induced collagen-induced arthritis (CIA) [5]. Therefore, TFA might be a promising strategy for HBV-positive RA patients.

## HBV vaccines

Vaccination remains the most effective strategy for preventing HBV infection. The development and implementation of prophylactic HBV vaccines not only protects against primary viral infection but may also reduce the risk of subsequent HBV-associated autoimmune complications. Therapeutic HBV vaccines, which aim to restore or enhance virus-specific immune responses, have demonstrated promising potential for augmenting antiviral immunity, particularly in chronically infected individuals with immune exhaustion. For example, TherVacB effectively elicits HBV-specific B cell responses and activates both helper and effector T cells through the presentation of HBsAg and HBcAg [81,82].

## Targeting metabolic therapies

In HBV infection, viral persistence is closely associated with the metabolic reprogramming of hepatocytes and immune cells, which collectively facilitate immune evasion and compromise antiviral responses. Similarly, in RA, hyperactive immune cells exhibit increased glycolysis, which contributes to persistent inflammation and joint degradation. Targeting dysregulated metabolic pathways, such as glycolytic flux or lipid metabolism, presents a promising strategy for restoring immune homeostasis. Metabolic rebalancing has the potential to rejuvenate exhausted antiviral T cells in HBV while simultaneously suppressing hyperactive, autoreactive immune populations in RA. Therefore, therapeutic regimens that incorporate metabolic modulators alongside direct-acting antivirals and immunoregulatory agents may offer synergistic advantages. These multidimensional strategies have the potential to improve viral clearance, reduce autoimmune exacerbations, and diminish long-term tissue damage.

## Conclusion and prospect

The emerging link between HBV infection and RA suggests a potential pathogenic role for HBV in promoting joint inflammation through a liver-joint axis. Current evidence highlights several key mechanisms underlying this inter-organ crosstalk, including fibroblast activation, immunometabolic dysregulation, aberrant signaling (e.g. PI3K/AKT, JAK/STAT), and immune imbalance (e.g. Th17/Treg skewing). Notably, this review also implicates the hepatic microbiome in RA pathogenesis: beyond HBV and its metabolites, bacteria such as Klebsiella pneumoniae (*K. pneumoniae*) and their associated metabolites may similarly contribute to disease progression. Targeting shared metabolic pathways (e.g. glycolysis) or multi-system signaling axes offers dual therapeutic benefits for enhancing antiviral immunity while suppressing autoimmunity. However, the molecular mechanisms linking HBV, the hepatic microbiota, and RA remain incompletely defined, particularly regarding the crosstalk between viral/bacterial pathogens and host metabolic-immune networks.

Future research should focus on four key areas: 1) Mapping virus-host-microbiome interactions using multi-omics (genomics, metabolomics, microbiomics) to identify novel pathogenic drivers at the liver-joint interface; 2) Elucidating immunometabolic crosstalk between hepatic inflammation (e.g. HBV-induced steatosis) and joint pathology, including the role of bacterial metabolites (e.g. Kp-derived molecules) in amplifying synovial inflammation; 3) Constructing systems-level models of the liver – joint axis to simulate pathogen-host-microbiome interactions and guide precision therapies; 4) Exploring phage-based interventions, such as screening bacteriophages targeting RA-associated pathogens (e.g. K. pneumoniae), to disrupt microbial-driven inflammation and offer innovative therapeutic strategies. Deeper mechanistic insight into HBV-RA comorbidity, integrating viral, microbial, and host metabolic factors, may ultimately support the development of more effective, multi-pronged interventions for managing systemic immune dysregulation in chronic inflammatory diseases.

## Data Availability

Data sharing is not applicable to this article as no data were created or analyzed in this study.
